# Personalized Risk Assessment for Taxane-Induced Hypersensitivity Reactions: A Systematic Review and Meta-Analysis

**DOI:** 10.3390/jpm15010002

**Published:** 2024-12-24

**Authors:** Hyun Jin Park, Minoh Ko, In-Wha Kim, Jung Mi Oh

**Affiliations:** 1 College of Pharmacy and Research Institute of Pharmaceutical Sciences, Seoul National University, Seoul08826, Republic of Korea; parkhj@kmu.ac.kr (H.J.P.); minovill@snu.ac.kr (M.K.); iwkim@snu.ac.kr (I.-W.K.); 2 College of Pharmacy, Keimyung University, Daegu 42601, Republic of Korea

**Keywords:** taxane, hypersensitivity reaction, risk factor, personalized risk assessment, systematic review, meta-analysis

## Abstract

**Background/Objectives:** Taxanes, including paclitaxel and docetaxel, are widely used in cancer treatment but frequently cause hypersensitivity reactions (HSRs), disrupting treatment continuity. This meta-analysis aimed to identify consistent risk factors for taxane-induced HSRs to support personalized risk assessments and optimize therapeutic outcomes. **Methods:** This systematic review and meta-analysis followed the PRISMA guidelines and was registered with PROSPERO (CRD42023476738). Comprehensive literature searches were conducted up to 30 June 2024. The quality of the studies was assessed using the Newcastle-Ottawa Scale. Data were synthesized to calculate pooled odds ratios (ORs) and 95% confidence intervals (CIs), using fixed or random effects models. **Results:** A total of 18 studies of moderate or higher quality were included, involving 8333 patients. The incidence of HSRs ranged from 3.0% to 33.1%. Risk factors assessed included history of allergy, obesity, postmenopausal state, ovarian cancer, and H2 receptor antagonist (H2RA) premedication. A history of allergy was identified as a potential risk factor with marginal significance (OR 1.85, 95% CI 0.97–3.54, *p* = 0.06), while H2RA premedication, ovarian cancer, and female sex were not significantly associated with HSR risk. Substantial heterogeneity was observed for obesity (I^2^ = 57.71%, *p* = 0.069) and postmenopausal status (I^2^ = 78.98%). **Conclusions:** This study highlights the complex nature of taxane-induced HSRs and emphasizes the need for personalized risk assessments. While a history of allergy is a potential risk factor, heterogeneity across other factors underscores the importance of individualized approaches. Clinicians should tailor strategies to balance the benefits of taxane therapy with patient-specific risks to improve clinical outcomes.

## 1. Introduction

Cancer remains one of the leading causes of death worldwide [1]. Among the various types of cancer, breast and lung cancer exhibit the highest incidence and mortality rates [2]. Taxanes, such as docetaxel and paclitaxel, exert their cytotoxic effects by binding to β-tubulin in microtubules, thereby preventing tubulin disassembly and ultimately arresting the cell cycle [3]. These agents are extensively utilized in the treatment of diverse cancers, including breast, ovarian, lung, head and neck, Kaposi sarcoma, gastric, and gynecological cancers. Taxanes are pivotal in the treatment of malignancies either as monotherapy or in combination with other agents [4,5]. Despite their broad clinical effectiveness, repeated use of taxane-based chemotherapy raises concerns about increased incidences of hypersensitivity reactions (HSRs), which can lead to premature treatment discontinuation and reduced overall efficacy [6].

Taxanes are associated with HSRs, which range from mild cutaneous reactions to severe, potentially life-threatening anaphylaxis, including bronchospasm, hypotension, and angioedema [7]. HSRs occur in approximately 30–50% of patients who do not receive premedication, typically during the first or second infusion [8,9,10,11]. To manage this risk, boxed warnings on paclitaxel and docetaxel package inserts emphasize the need for premedication and advise against rechallenging patients who have experienced severe HSRs [12,13]. These adverse reactions are influenced by the physicochemical properties of taxanes, such as high lipophilicity and low water solubility, necessitating the use of solvents like Cremophor EL and polysorbate 80, which have been implicated in severe reactions [14]. Mechanistically, these reactions may involve several potential pathways: immunoglobulin E (IgE) or immunoglobulin G (IgG)-mediated activation of mast cells or basophils triggered by the taxane or solubilizer, direct non-IgE-mediated activation of basophils or mast cells, or IgG-mediated activation of the complement system via immune complexes [11,15,16].

To mitigate these risks, premedication strategies including glucocorticoids and antihistamines are used, reducing the incidence of mild to moderate HSRs to 5–10%, though severe HSRs persist [7,17,18]. Albumin-bound paclitaxel, developed to eliminate solvent-related risks, has demonstrated a significantly lower incidence of HSRs. The Food and Drug Administration (FDA) does not recommend premedication for nab-paclitaxel due to its favorable safety profile [19]. However, reports of HSRs to nab-paclitaxel, though rare, have still been reported, and suggest that treatment tolerance may diminish with repeated infusions. To further alleviate these risks, novel taxane delivery systems are being explored to bypass intravenous administration and associated IgE-mediated infusion reactions. Recent advancements in oral formulations aim to enhance paclitaxel’s bioavailability and provide an alternative to the traditional intravenous route. These include liposomes, polymeric micelles such as paclitaxel-pegylated poly(anhydride) nanoparticles, paclitaxel-loaded CMCS-Qu polymeric micelles, and paclitaxel nanoemulsions. Additionally, transdermal formulations have been developed using microemulsions and elastic liposomes. Nanovesicles and lipid nanocarriers are also being explored for intranasal or pulmonary delivery systems [20]. Furthermore, recent advances in HSR desensitization protocols have provided effective strategies for reducing HSRs in taxane-treated patients. These protocols, which involve gradual dose escalation under controlled conditions to establish temporary tolerance, have been associated with improved overall survival outcomes [21]. However, desensitization protocols require extended infusion times and intensive labor resources, making it burdensome for both patients and healthcare providers. Additionally, despite these protocols, there remains a risk of breakthrough HSRs during the desensitization process, and the tolerance achieved is only temporary [7].

Genetic predispositions, such as variations in drug metabolism or immune response pathways, may also contribute to individual susceptibility to taxane-induced HSRs [11,15,16]. This variability underscores the limitations of generalized premedication protocols and highlights the need for a more tailored approach through personalized risk assessment. Recently, a clinical prediction model for taxane-induced HSRs has been developed, incorporating features such as age, performance status, prior history of taxane HSRs, medication allergy history, comorbidities, actual dose of taxane, and absence of premedication, among others [22]. While the model demonstrated a specificity around 75%, its sensitivity was relatively low at 57.07%. To develop a personalized prediction model with improved accuracy, it is crucial to investigate and identify the risk factors associated with taxane-induced HSRs. While previous studies have explored potential mechanisms and risk factors, the evidence is often inconsistent and lacks robustness. Risk factors such as age, allergy history, taxane dosage, administration time, and cancer types have been reported inconsistently across previous studies [18,21,23,24,25,26,27,28,29,30,31,32,33,34,35,36,37,38,39,40,41,42]. This gap between studies highlights the need for a comprehensive synthesis of the existing data to clarify the epidemiology of taxane-induced HSRs and identify consistent risk factors for personalized risk assessment. In addition, while the novel formulations are under development, their path to commercialization is often lengthy. To optimize the use of existing formulations and mitigate patient risks, conducting comprehensive risk factor analyses and synthesizing robust evidence is essential.

While taxanes are essential for cancer treatment, the considerable risk of HSRs they present impedes clinical utilization. Comprehensive research is imperative to thoroughly grasp the epidemiology of taxane-induced HSRs, identify consistent risk factors, and implement personalized premedication and desensitization strategies based on individual risk profiles. By providing evidence-based insights, the study seeks to inform optimized personalized clinical strategies, enhance patient safety, and ensure the effective administration of taxane-based treatments. This systematic review/meta-analysis aims to consolidate existing knowledge on taxane-induced HSRs by identifying consistent risk factors and their potential impact on clinical outcomes.

## 2. Materials and Methods

### 2.1. Study Protocol

This research strictly followed the Preferred Reporting Items for Systematic Reviews and Meta-Analyses (PRISMA) statement [43]. The study protocol was prospectively recorded in the PROSPERO database (CRD42023476738).

### 2.2. Search Strategy and Eligibility Criteria

Two authors (H.P. and M.K) independently searched the literature, screened title and abstracts for eligibility, and selected studies for inclusion. Any disagreements between the authors were resolved through joint discussions to ensure consensus. If consensus could not be reached, a third reviewer (I. K.) mediated the discussion to finalize decisions. A comprehensive literature search was conducted across multiple databases, including MEDLINE, Pubmed, EMBASE, and Web of Science, to identify relevant studies published up to 30 June 2024. The search terms included: (“docetaxel” OR “paclitaxel” OR “cabazitaxel” OR “taxan*”) AND (“risk*” OR “factor*” OR (“gene*” OR “genotyp*” OR “allele*” OR “polymorphism*” OR “pharmacogene*” OR “variant*” OR “haplotype*” OR “genome*” [Title/Abstract] OR “SNP” OR “HLA”)) AND (“hypersensitivit*” OR “HSR” OR “infusion related*” OR “infusion reaction*” OR “allerg*” OR “anaphyla*” OR “SCAR*” OR “toxic epidermal necrolysis*” OR “SJS” OR “stevens johnson syndrome*” OR “DRESS*” OR “drug reaction with eosinophilia systemic symptoms*”). The detailed search strategy can be found in Supplementary S1, and the definitions of risk factors in studies included in the meta-analysis are provided in Supplementary S2.

The inclusion criteria for the studies in this review were as follows: (1) studies involving adult cancer patients aged 18 years and older receiving taxane-based chemotherapy with docetaxel, paclitaxel, or cabazitaxel; (2) studies reporting on the risk factors for taxane-based HSRs; (3) observational studies including cohort, case-control, and cross-sectional designs; (4) studies published in English; and (5) studies reporting odds ratio (OR), hazard ratio (HR), and relative risk (RR) with the corresponding 95% confidence interval (CI), or reported data for HSR events for statistical calculation. The exclusion of pediatric populations was due to the limited use of taxane-based chemotherapy in children, as taxanes are primarily indicated for adult malignancies, as well as the potential differences in HSR presentation and immune response between children and adults. Studies were excluded if they met any of the following criteria: (1) abstracts without full text; (2) case reports, case series, or review articles; (3) studies focusing solely on the incidence of HSRs without risk factor analysis; (4) studies involving pediatric populations; (5) studies with incomplete or unclear data on risk factors or HSR outcomes; and (6) studies reporting risk factors for combination chemotherapy, without focusing on paclitaxel or docetaxel-specific risk factors.

### 2.3. Data Extraction and Quality Assessment

Two independent reviewers (H.P. and M.K.) independently extracted data using a standardized form for each study. The extracted data included the first author’s name, year of publication, country, study design, study population, period of study, sample size, and patient demographics such as age and sex. Additionally, details about the type of cancer, chemotherapy regimens, and specific taxane usage information, such as cycle number, cumulative dose, and the specific type of taxane HSRs observed, were collected. Information on the definition and incidence of HSRs as well as risk factors with their respective effect sizes (e.g., OR, RR, HR) and 95% CI were also documented. Any disagreements were resolved through discussion or consultation with a third reviewer (I.K.).

The risk of bias for the included observational studies was evaluated using the Newcastle-Ottawa Scale (NOS), which is a specific scale used to assess the quality of non-randomized studies in meta-analyses, applying criteria tailored to the specific study type, whether cohort or case-control, to ensure appropriate assessment. To assess publication bias, Egger’s and Begg’s tests and funnel plots were employed.

### 2.4. Meta-Analysis

In this study, meta-analyses were conducted exclusively for studies that met the criteria for quantitative synthesis. Studies were excluded from pooled analysis if they exhibited discrepancies in reported outcomes (e.g., odds ratios versus hazard ratios) or inconsistencies in variable definitions and characteristics, ensuring methodological rigor and reliability. All identified risk factors, including demographic, clinical, and genetic variables, were initially treated with equal weight during the data synthesis and meta-analysis. This method was used to conduct a systematic evaluation of the potential associations between a variety of risk factors and taxane-induced HSRs. While certain factors, such as genetic polymorphisms, may have distinct mechanistic implications, our methodology was designed to identify potential associations among variables without pre-defining a hierarchy of significance. The risk factors for taxane-induced HSRs were synthesized using ORs with 95% CIs. The risk factors with an OR greater than 1 were considered indicative of an increased risk for HSRs. The heterogeneity among the included studies was assessed using Cochran’s Q test and I^2^ statistics [44,45]. An I^2^ greater than 50% or *p*-value less than 0.10 was considered to indicate statistically significant heterogeneity. In cases of statistically significant heterogeneity, a random-effects model was applied for meta-analysis; otherwise, a fixed-effects model was applied [46]. Subgroup analyses were conducted for groups containing more than two studies to explore the effects within different risk factor subgroups. Sensitivity analyses were conducted to evaluate the robustness of the results by sequentially excluding individual studies and observing the impact on overall effect estimates. All statistical analyses and visualizations were performed using Comprehensive Meta-Analysis Software (CMA) version 4 (Biostat, Inc., Englewood, NJ, USA). A two-sided *p*-value of less than 0.05 was considered statistically significant.

## 3. Results

### 3.1. Study Selection

**Figure 1:**
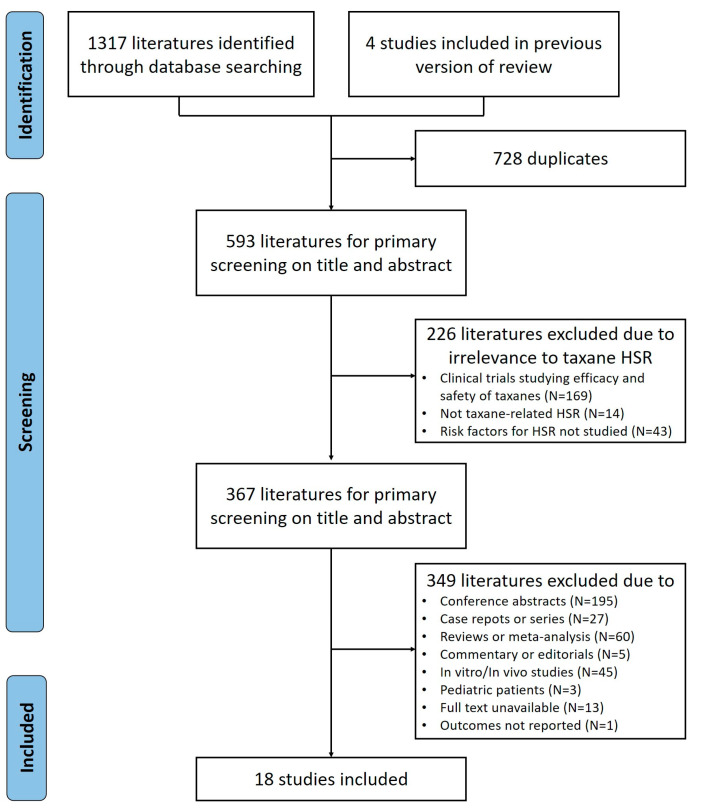
Flow chart of the literature search and study selection process.

A comprehensive literature search was conducted across multiple databases including MEDLINE, Pubmed, EMBASE, and Web of Science to identify studies pertaining to taxane-induced HSRs. The initial search yielded 1321 papers, four of which were included in a previous review [47]. Initially, 728 duplicate records were removed to ensure accuracy. Further screening of titles and abstracts resulted in the exclusion of 226 publications irrelevant to taxane-induced HSRs. Among the excluded studies were 169 studies that focused solely on the efficacy and safety of taxanes without addressing HSRs, 14 studies not related to taxane-induced HSRs, and 43 studies that did not explore risk factors for HSRs. Exclusions were also made based on study types that did not support robust data analysis, including in vitro or in vivo studies, studies involving pediatric populations, and studies with unavailable full texts or insufficient outcome reporting. Figure 1 illustrates the study selection process in detail.

### 3.2. Study Characteristics

Table 1 details the characteristics of the 18 included studies, spanning a broad spectrum of global region and oncological diagnoses. These studies comprise seven case-control studies, six retrospective cohort studies, two retrospective chart analyses, a post-hoc analysis, and two prospective studies, collectively involving 8333 cancer patients, with sample sizes ranging from 76 to 3181 participants. The majority of the research focused on breast and gynecological cancer, though other studies addressed various cancer types, including gastrointestinal, genitourinary, head and neck, and lung cancers. The participant age across studies varied, with ages ranging from 27 to 84 years, and mean or median ages typically falling in the early to mid-fifties. The studies prominently featured female patients, particularly in those concerning breast and gynecological cancers. Methodological quality was rigorously evaluated using the NOS criteria, categorizing four studies as moderate quality and fourteen as high quality.

Among the eighteen studies included in this review, eight were conducted in the Asia-Pacific region. One study [30], a post-hoc analysis of a phase III clinical trial, included patients from New Zealand and Australia, along with European countries. In total, seven studies were conducted in European regions, while four studies were conducted in North and South America, including the post-hoc analysis [30].

**Figure 2:**
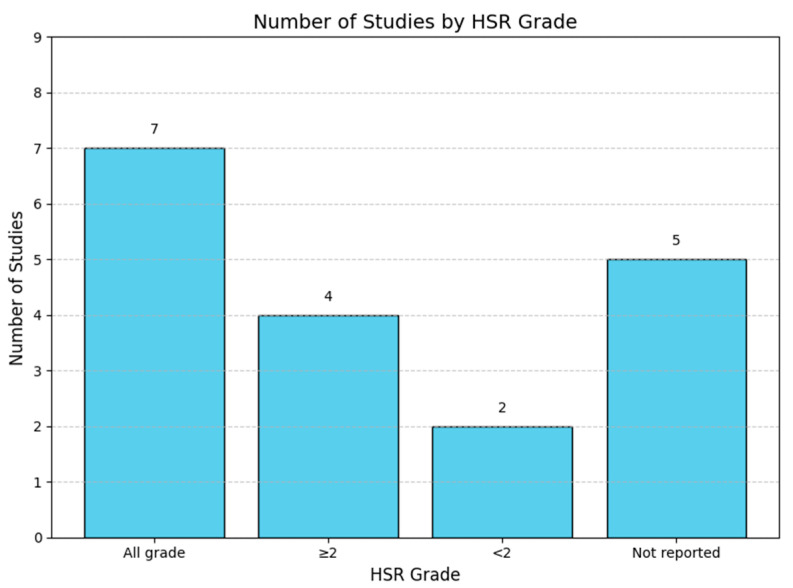
Number of studies by HSR grade.

**Figure 3:**
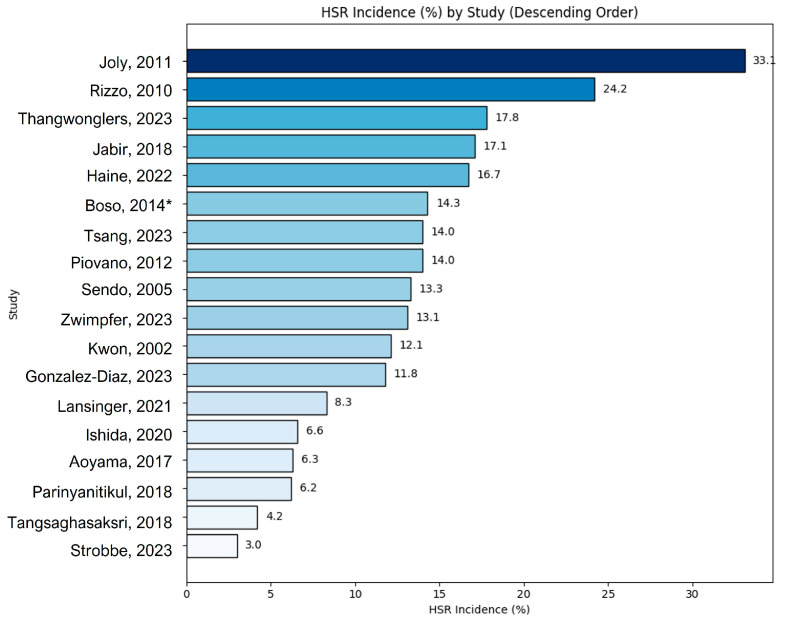
Incidence of hypersensitivity (HSR) by each study. (HSR: hypersensitivity, * This is data on docetaxel) [18,21,24,25,27,28,30,31,33,34,35,36,37,38,39,40,41,42].

Studies from the Asia-Pacific region [19,25,29,34,35,36,37] predominantly recruited patients with breast and gynecological cancers. These studies assessed both demographic factors, such as age, body weight, postmenopausal status, and cancer stage, and clinical factors like history of allergies, premedication protocols, white blood cell counts, dose/administration schedules, and diluent volumes. Notably, a Japanese study identified the genetic polymorphism ABCB1 3435C>T as a potential risk factor for taxane-induced HSRs. In European studies [18,21,24,26,32,33], patients with various solid tumors, including breast and gynecological cancers, were recruited. CYP3A4 genetic polymorphisms were consistently highlighted as a factor associated with taxane-induced HSRs. Other assessed risk factors included premedication protocols, postmenopausal status, history of allergies, BMI, age, and the timing of taxane administration, specifically during the first three injection cycles. In North and South America, four studies [14,22,28,31] involved patients with breast and gynecological cancers as well as other diverse cancer types. Factors such as atopy history, routes of administration, and doses of prophylactic corticosteroids were evaluated. One retrospective cohort study from the United States included patients of various racial backgrounds; however, the results of an adjusted multivariate analysis indicated that race was not associated with an increased risk of taxane-induced HSRs. Paclitaxel was frequently studied as the primary culprit for HSR, although five studies also examined docetaxel-induced HSRs. Most studies involved regimens that combined taxanes with other chemotherapeutic agents, such as platin salts or monoclonal antibodies such as trastuzumab. Risk factors for HSRs were extensively analyzed, considering variables such as sex, age, previous platinum exposure, allergy history, metastatic status, and specific drug combinations. The incidence rates of HSRs varied widely, ranging from a low of 3.0% to a high of 33.1% (Table 2). The cumulative taxane doses were reported in only two studies: a retrospective cohort study [18] and a case-control study [35]. In the retrospective cohort study, the cumulative dose for the cohort was reported as 1503 mg (median, interquartile range [IQR]: 1042–1691). In the case-control study, the cumulative dose for the case group was 267.54 ± 29.14 mg (mean ± standard deviation [SD]), while the control group had a similar cumulative dose of 267.41 ± 37.91 mg. The distribution of studies by HSR grade shows that most studies reported on all grades of HSRs (*n* = 7), followed by studies focusing on grade ≥ 2 HSRs (*n* = 4), grade < 2 HSRs (*n* = 2), and those that did not report specific HSR grades (*n* = 5) (Figure 2). HSR incidence varied across studies, with the highest reported in Joly et al. [35] at 33.1%, followed by Rizzo et al. [38] at 24.2%, and the lowest in Strobbe et al. [27] at 3.0% (Figure 3).

### 3.3. Quality Assessment

The risk of bias for each study using NOS is presented in Supplementary S3. The NOS assessment demonstrates that the majority of the included studies are of good quality, with only a few rated as moderate. The funnel plots in Supplementary S4 indicate a well-balanced dataset with no obvious signs of publication bias.

### 3.4. Heterogeneity

Assessment of heterogeneity among various studies concerning risk factors for taxane-induced HSRs was conducted thoroughly. Significant heterogeneity was noted in the postmenopausal state (I^2^ = 78.98%, *p* = 0.003, τ^2^ = 0.52), analyzed using a random effects model due to substantial variability in study outcomes. Moderate heterogeneity was observed for factors such as ovarian cancer (I^2^ = 47.73%, *p* = 0.167, τ^2^ = 0.10) and obesity (I^2^ = 57.71%, *p* = 0.069, τ^2^ = 0.52), reflecting diverse effects across studies. In contrast, risk factors like histamine 2 (H2) blocker premedication, including specific analysis for ranitidine, demonstrated negligible heterogeneity (I^2^ < 0.1%, *p* > 0.900, τ^2^ < 0.01), indicating consistent results across studies, and these were thus analyzed using a fixed effect model. These findings underscore the importance of considering individual study characteristics and the influence of specific risk factors on outcomes in taxane hypersensitivity research.

### 3.5. Risk Factors Associated with Taxane HSRs

#### 3.5.1. History of Allergy

**Figure 4:**
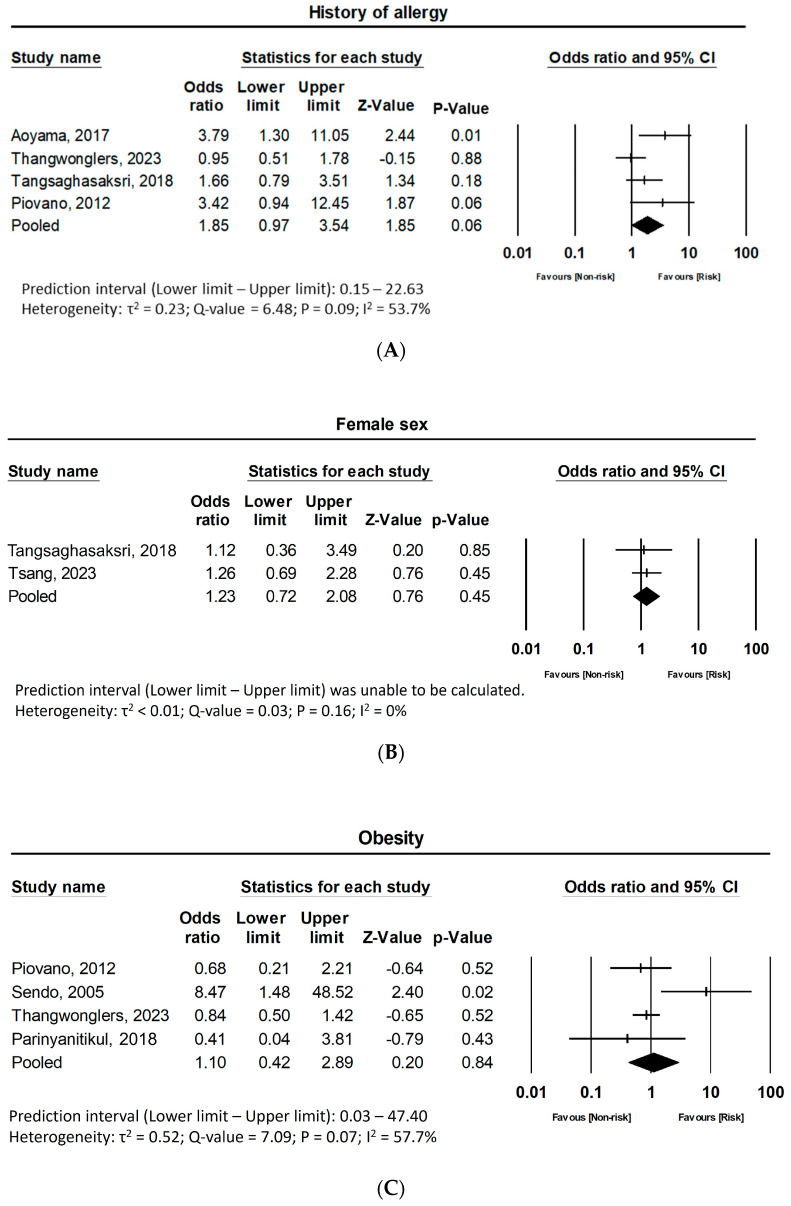

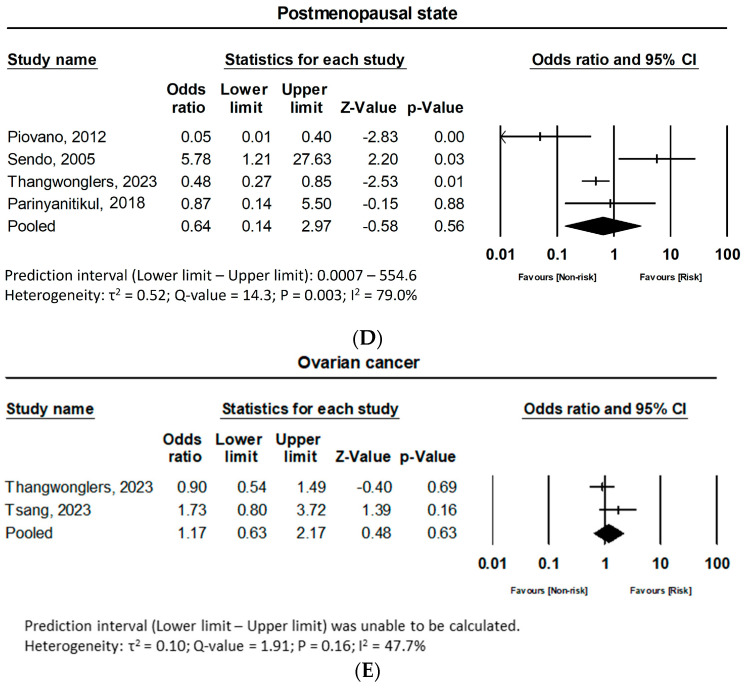
Association between patient characteristics and taxane-induced hypersensitivity reactions. (**A**) History of allergy; (**B**) Female sex; (**C**) Obesity; (**D**) Postmenopausal state; (**E**) Ovarian cancer. CI: confidence interval. [28,30,37,39,40,41,42].

Five studies [30,33,37,40,41] from this review evaluated the history of allergy, defining it as systemic allergic reactions to radiocontrast media or drugs. Notably, Joly et al. [35] reported the OR for the history of systemic HSRs using data from the CALYPSO trial, which included patients not treated with taxanes. Consequently, these data were excluded from the pooled analysis. A pooled analysis of the remaining studies yielded an overall OR of 1.85 (95% CI 0.97–3.54, Z = 1.85, *p* = 0.064), indicating a marginally non-significant increase in risk for patients with a history of allergy. The forest plot is featured in Figure 4A. Gonzalez-Diaz et al. [33] evaluated the association between sensitization to environmental or food allergens and the history of atopy with taxane-induced HSRs. While sensitization to allergens showed no significant impact, both personal and family history of atopy were associated with increased risk of taxane HSRs (OR 5.00, 95% CI 1.07–24.19 and OR 4.60, 95% CI, 1.02–21.33, for personal and family history, respectively). However, the limited number of patients included in the study calls for caution, as this affects the statistical power and robustness of the findings. Leave-one-out analysis also revealed insignificant association (Supplementary S5(A)).

#### 3.5.2. Age

Five studies assessed age as a risk factor for hypersensitivity reactions, with two studies treating age as a continuous variable and the remaining four using age cut-offs at 50, 48, 70, and 54.5 years to evaluate its association with increased risk as a dichotomous variable. Due to differences in definitions and heterogeneity, a pooled analysis could not be performed. Two studies conducted in Japan showed contrasting results. The study by Aoyama et al. [30] reported that younger age, under 50, was associated with a statistically significant increase in the risk of HSRs by 6.31-fold (OR 6.31, 95% CI 2.65–15.7). In contrast, the study by Ishida, et al. [34] did not demonstrate any association between being under 48 years of age and an increased risk of HSR occurrence (OR 3.47, 95% CI 0.94–13.40). Still, other studies have consistently shown a trend that younger age is associated with an increased risk of HSRs. For instance, the study by Joly et al. [35] found that being 70 or above was associated with a 55% decreased risk of HSRs (OR 0.450, 95% CI 0.255–0.794). Additionally, Thangwonglers et al. [41] demonstrated a significant association between an increased risk of HSRs and age below 54.5 years (OR 2.35, 95% CI 1.41–3.93).

#### 3.5.3. Other Demographic Factors: Sex and Race

Three studies assessed sex as a risk factor for taxane-induced HSRs. Tsang et al. [28] and Tangsaghasaksri et al. [40] evaluated the risk for female patients compared to male, finding no significant association (OR 1.26, 95% CI 0.69–2.28 and OR 1.12, 95% CI 0.36–3.49, respectively). The pooled results of these two studies showed no association between HSRs and female sex (OR 1.23, 95% CI 0.72–2.08) (Figure 4B). Conversely, Lansinger et al. [36] conducted a retrospective cohort study and identified a significant association, with female sex linked to an increased risk of taxane-induced HSRs (hazard ratio [HR] 1.26, 95% CI 1.09–1.46). Additionally, Lansinger et al. [36] was the only study to explore whether race could be a contributing factor to taxane-induced HSRs, though no significant associations were found.

#### 3.5.4. Obesity

From the included studies, three studies evaluated the association between obesity and increased risk of taxane hypersensitivity. All three studies homogenously defined obesity as a body mass index (BMI) more than 25 kg/m^2^. The pooled analysis assessed the influence of obesity on the incidence of taxane-induced HSR, and the forest plot is illustrated in Figure 4C. A combined analysis of four studies yielded a pooled OR of 1.10 (95% CI 0.42–2.89, Z = 0.20, *p* = 0.84), demonstrating that obesity does not significantly impact the risk of HSR. Sensitivity analysis maintained the results from the pooled analysis, and the forest plot results are presented in Supplementary S5(B) (OR 0.79, 95% CI 0.49–1.26, Z = −1.00, *p* = 0.32).

#### 3.5.5. Postmenopausal State

Three studies investigated the postmenopausal state as a risk factor for taxane-induced HSRs. Piovano et al. [37] and Thangwonglers et al. [41] reported a significant association between postmenopausal status and decreased risk of taxane-induced HSRs (OR 0.05, 95% CI 0.01–0.40 and OR 0.48, 95% CI 0.27–0.85, respectively). Conversely, Sendo et al. [39] indicated a positive association, noting an increased incidence of HSRs among postmenopausal women. A forest plot summarizing data from four studies on the impact of postmenopausal status is displayed in Figure 4D. The pooled OR was 0.64 (95% CI 0.14–2.97, Z = −0.58, *p* = 0.56), suggesting a non-significant trend toward reduced hypersensitivity reactions in postmenopausal patients. Removing the most heterogeneous study showed similar results (Supplementary S5(C)).

#### 3.5.6. Cancer Type: Ovarian Cancer

Two studies [28,41] that explored ovarian cancer as a risk factor for taxane-induced HSRs were incorporated into the meta-analysis, with results displayed in Figure 4E. Using a random effects model, the analysis indicated that ovarian cancer did not significantly alter the risk of taxane-induced hypersensitivity reactions (HSRs) compared to other oncologic conditions, with a pooled OR of 1.17 (95% CI: 0.63–2.17, Z = 0.48, *p* = 0.63).

#### 3.5.7. Premedication Regimens: H2 Receptor Antagonist (H2RA) Premedication

**Figure 5:**
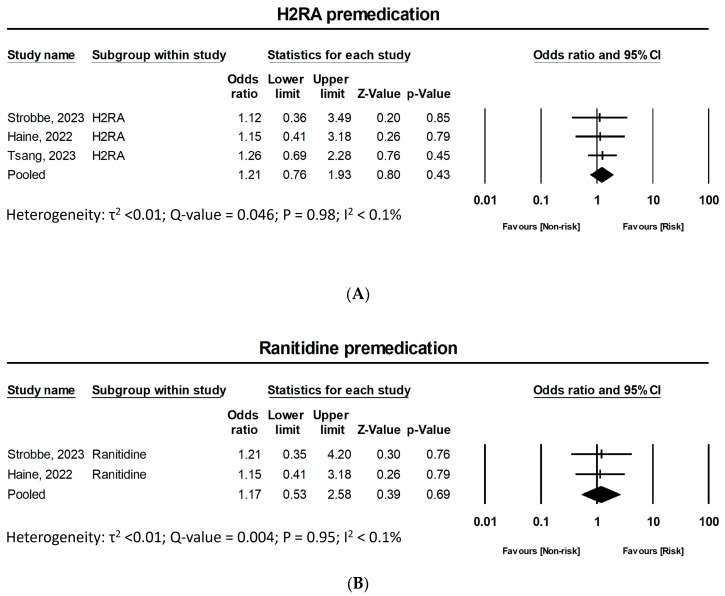
Association between H2RA premedication and taxane-induced hypersensitivity reactions. (**A**) H2 receptor antagonist premedication; (**B**) Ranitidine premedication subgroup analysis. CI: confidence interval, H2RA: Histamine H2 receptor antagonist [24,27,28].

Three studies assessed the association between the premedication of H2RA and the occurrence of taxane-induced HSR. Strobbe et al. [27] and Tsang et al. [28] included both famotidine and ranitidine in their assessment, while Haine et al. [24] specifically focused on ranitidine, and Strobbe et al. [27] also conducted a separate subgroup analysis focused solely on ranitidine. A pooled analysis of these three studies was performed to determine the effect of H2-blocker premedication versus no premedication on taxane-induced HSR. The results yielded a pooled OR of 1.21 (95% CI 0.76–1.93, Z = 0.80, *p* = 0.43), demonstrating a non-significant risk associated with H2-blocker premedication. After excluding Tsang et al. [28] from the analysis, the pooled the results showed a pooled OR of 1.17, indicating no significant association between ranitidine and taxane HSRs (95% CI 0.53–2.58, Z = 0.39, *p* = 0.69). The forest plot is featured in Figure 5.

#### 3.5.8. Genetic Polymorphisms

In breast cancer patients, genetic variants were investigated for their association with docetaxel hypersensitivity. The study conducted by Boso et al. [31] highlighted that variants CYP3A4*1B (rs2740574) and CYP3A5*1 (rs776746) were linked to an increased frequency of infusion-related reactions, with statistical significance (*p* = 0.01 and *p* = 0.036, respectively). Conversely, the research by Rizzo et al. [38] identified the CYP1B1*3 variant (rs1056836) as being associated with a reduced risk of hypersensitivity, with an OR of 0.14 and a 95% CI of 0.049–0.38, indicating a protective effect against adverse reactions.

### 3.6. Sensitivity Analysis

Sensitivity analyses were conducted to evaluate the robustness of the pooled results by excluding individual studies. The findings indicated that the results remained consistent across all risk factors. For history of allergy, obesity, and postmenopausal status, the overall trends were consistent with the main analysis, confirming the robustness and the reliability of the pooled results (Supplementary S5).

## 4. Discussion

This comprehensive meta-analysis of 18 studies involving 8333 patients provides important insights into the risk factors for taxane-induced HSRs, supporting personalized risk assessments that enable clinicians to predict individual susceptibility and personalize premedication strategies based on individual risk factors, ultimately improving treatment safety and efficacy. Our key findings include: (1) a marginally significant association between history of allergy and increased HSR risk; (2) substantial heterogeneity in the effects of factors such as obesity and postmenopausal state; (3) no significant association for factors including sex, cancer type, and H2RA premedication; and (4) a potential role of genetic polymorphisms, though limited by the small number of studies.

Risk factors associated with taxane-induced HSRs have been the subject of numerous studies, including those that were incorporated into our meta-analysis and those that were not. Among the studies, a recent retrospective analysis by Trager et al. [48] identified age, race, and a prior history of allergy as significant risk factors for immediate paclitaxel-induced HSRs. Sa-Nguansai et al. [22] identified eleven predictors for paclitaxel HSRs and developed a risk prediction model based on these risk factors. Additionally, numerous studies have examined the impact of various premedication strategies and desensitization protocols on the incidence of taxane-induced HSRs. Recent research on premedication strategies includes Hutchinson et al. [49], who reported that adding cetirizine to the premedication regimen reduced the severity of grade 3–4 HSRs; Xiao et al. [50], who investigated the optimal dose of dexamethasone for premedication therapy; Symons et al. [51], who evaluated the effectiveness of a modified desensitization protocol as primary prophylaxis in patients treated with paclitaxel. Incorporating the available studies by using a comprehensive search strategy, this study offers significant insights into the clinical and demographic factors that may contribute to the probability of developing HSRs to paclitaxel and docetaxel-based chemotherapy. While pharmacogenomic risk factors may be the most relevant risk factor to HSRs due to their mechanistic implications, the limited availability of relevant studies for analysis restricted a comprehensive meta-analysis of these factors. In the included studies, the mean incidence of paclitaxel-induced HSRs was 12.29% (±7.26), while for docetaxel, it was 16.63% (±7.86), observed in various populations from countries including Japan, Spain, Mexico, Netherlands, Malaysia, Canada, the United States, Italy, France, Thailand, and Switzerland [18,21,24,25,27,28,30,31,33,34,35,36,37,38,39,40,41,42]. In our pooled analysis, we identified 59 potential risk factors; however, only six—supported by data from more than two studies—were analyzed in detail. The key findings surrounding each of the identified risk factors for taxane HSRs are discussed below. Other risk factors that could not be synthesized due to lack of research include race and genetic polymorphisms. Additionally, with regard to age, it was not possible to synthesize the results because the age definitions for the analyses were different for each study.

Our analysis revealed that patients with a history of allergies had a marginally increased risk of taxane HSRs, consistent with previous studies linking atopic conditions with a higher likelihood of drug-induced HSRs to various drugs [33,52,53,54]. Although Aoyama, et al. [30] identified a strong association (OR 3.79, 95% CI 1.30–11.05) between allergy history and increased HSR risk, subsequent studies by Thangwonglers et al. [41] and Tangsaghasaksri et al. [40] did not demonstrate significant associations. The wide prediction interval (0.15 to 22.63) indicates substantial heterogeneity, suggesting that the effect of allergy history on HSRs may vary across populations. This variability could stem from differing definitions and inclusion of allergy history; Aoyama et al. [30] considered only medical drugs and contrast agents, while Piovano et al. [37] and Thangwonglers et al. [41] also included environmental and food allergens. This indicates that although allergy history may not always reach statistical significance, it could still have clinical importance, especially in patients with documented allergic reactions to other therapeutic drugs. Clinicians should therefore consider this risk factor when assessing and personalizing patient risk for taxane HSRs. Specifically, when integrating multiple risk factors into clinical decision-making, the severity and specificity of the allergic history should be carefully prioritized. Patients with a documented history of severe allergic reactions to therapeutic drugs may require heightened monitoring and premedication strategies due to the increased potential for HSRs with taxanes. Clinicians should weigh risk factors holistically, prioritizing interventions based on the individual patient’s risk profile.

Regarding obesity, our findings were inconclusive, with significant heterogeneity across studies. The variability in findings regarding obesity as a risk factor for taxane-induced HSRs is particularly noteworthy and may be attributed to variations in the criteria used to define obesity across the included studies, characteristics of the population studied, and definition of HSRs. Among the four studies analyzed, three of the four studies included in this analysis were conducted in Asian populations and one in Italy. Additionally, three studies defined obesity as BMI > 25 kg/m^2^, while one defined it as BMI > 30 kg/m^2^. Sendo et al. [39] reported a significantly higher risk of HSRs (OR 8.47, 95% CI 1.48–48.52), while other studies showed no significant associations, contributing to the overall non-significant pooled outcome. The wide prediction interval (0.026 to 47.40) highlights considerable heterogeneity, suggesting that obesity’s effect on HSRs may differ across populations. Differences in study designs, including retrospective cohort study and case-control study, further contribute to the result variability. These findings imply that obesity may not consistently influence hypersensitivity outcomes, but its impact could be significant under specific populations or under certain conditions. Clinically, obesity is closely associated with the development of various cancers, and thus obesity as a possible risk factor for taxane HSRs necessitates close monitoring during taxane treatment [55]. In order to clarify the implications for clinical practice and facilitate the development of individualized strategies for the management of taxane HSR risk, future research should further examine population characteristics, study design, and definition differences.

Similarly, findings for the postmenopausal state varied in its association with HSRs risk. The wide prediction interval (0.001 to 554.64) for postmenopausal state suggests marked heterogeneity, indicating that the influence of postmenopausal status on HSRs may vary considerably in different clinical settings. This variability could be attributed to the differences in the characteristics of study populations, including ethnicity, hormonal profiles, and cancer types, as well as differences in study designs. This complexity calls for a careful interpretation, as postmenopausal status could have an important influence on hypersensitivity outcomes in certain subgroups, particularly in elderly women receiving taxane-based chemotherapy.

H2RA premedication, including famotidine and ranitidine, was not significantly associated with taxane HSRs, as reflected by ORs consistently close to unity in the studies. The prediction interval (0.36 to 3.49) indicates moderate heterogeneity, suggesting varying effects across different populations. The subgroup analysis focusing on ranitidine also showed non-significant results, emphasizing the need for further investigation into its potential impact on HSR risk.

This meta-analysis consolidates data from multiple observational studies, establishing a solid basis for future research and providing insights that could inform more personalized management of HSRs in clinical practice. Beyond statistical significance, the clinical importance of these findings must be considered. While the associations for some factors—such as allergy history, obesity, and postmenopausal status—did not consistently achieve strong statistical significance, they still hold clinical relevance in guiding personalized risk assessment and HSR management strategies. Understanding these factors allows clinicians to better predict which patients may be more susceptible to taxane-induced HSRs, enabling them to individualize premedication regimens and monitoring strategies. This personalized approach would potentially improve patient outcomes by reducing the risk of HSRs. Clinicians should recognize patient characteristics, such as a history of allergies, obesity, or postmenopausal status, and personalize treatment plans for more effective risk management in taxane-based chemotherapy.

To the best of our knowledge, this is the first study to synthesize the risk factors associated with taxane-induced HSRs, making a substantial contribution to the field by addressing the gaps in the literature and facilitating personalized risk assessment. We incorporated all relevant studies published up to the most recent data (30 June 2024), utilizing a comprehensive and strategic search methodology to ensure thorough coverage. This meta-analysis has several strengths, including its comprehensive scope, rigorous methodology adhering to PRISMA guidelines, and the large combined sample size. A notable strength of our study is the inclusion of a diverse set of populations and cancer types, enhancing the generalizability of our findings across various clinical settings. Additionally, our systematic approach facilitated the identification and analysis of several potential risk factors, providing a comprehensive perspective on the factors that may contribute to taxane HSRs. These findings are essential for clinicians aiming to implement personalized risk stratification, facilitating more precise and patient-specific premedication and management regimens based on individual risk profiles. This meta-analysis provides important insights into potential risk factors for taxane-induced HSRs while highlighting areas for further investigation. Although risk factors have not reached statistical significance across analyses, certain patient characteristics—such as a history of allergies, obesity, or postmenopausal status—may still play a critical role in the personalized management of HSRs in patients undergoing taxane-based chemotherapy. Clinicians should consider the diversity in individual patient risks when developing premedication strategies. Clinicians could incorporate the identified risk factors into premedication protocols by prioritizing a history of allergic reactions, such as enhancing monitoring or adjusting corticosteroid and antihistamine regimens for high-risk patients. Also, obesity and postmenopausal state should also be considered, with tailored approaches like dose modifications or closer observation to mitigate HSR risks. By integrating personalized risk factors and pharmacogenomic profiles, healthcare providers can enhance the prevention and management of HSRs, leading to more effective and safer taxane-based treatments.

Despite the comprehensive analysis, this meta-analysis has some limitations. The significant heterogeneity observed for several risk factors limits the conclusiveness of some findings. The reliance on observational studies introduces potential for confounding and bias, which could affect the results. Additionally, the limited number of studies available for certain risk factors, particularly genetic polymorphisms, restricts the robustness of these analyses. The varying definitions of HSRs across studies may have affected the comparability of results. Although subgroup analyses of taxane HSR risk factors by cancer type, geographic region, and taxane type would have provided more thorough risk factor results, the limited number of available studies impeded these analyses, highlighting the need for future research as more data becomes available. Considering the potential biological and genetic differences among ethnic groups, future research should investigate ethnicity-specific and pharmacogenomic risk factors and conduct analyses in more homogeneous cohorts to improve personalized risk assessments and refine individualized strategies. Moreover, subgroup analyses based on genetic and ethnic variations, along with further exploration of pharmacogenomic factors, would provide a deeper understanding of individual susceptibility to taxane HSRs. Prospective studies with larger, more diverse cohorts are needed to validate these findings, and explore the underlying mechanisms of taxane-induced HSRs. Comprehensively incorporating pharmacogenomics as a risk factor and personalizing chemotherapy premedication regimens will serve as crucial steps toward reducing the incidence and severity of HSRs, ultimately improving clinical outcomes in clinical practice.

## 5. Conclusions

In conclusion, this meta-analysis provides a comprehensive overview of risk factors for taxane-induced HSRs, highlighting the complex and multifactorial nature of this adverse event. While a history of allergy emerged as a potential risk factor, the heterogeneity in findings for other factors underscores the need for personalized risk assessment. Our results emphasize the importance of moving beyond one-size-fits-all approaches to patient management in taxane therapy. Clinicians should consider these findings in their decision-making processes, aiming for a balanced approach that considers both the potential benefits of taxane therapy and the individualized risk of HSRs. Future studies should aim to address the current gaps by investigating the impact of ethnicity-specific risk factors and the role of pharmacogenomics in achieving more stratified risk prediction. Ultimately, integrating these findings with other risk factors could provide more precise strategies for managing taxane therapy, thereby improving clinical outcomes for patients.

## Figures and Tables

**Table 1:** Summary of included study characteristics.

Author, Year	Country	Study Design	Culprit Drug	Oncologic Diagnosis	Number of Patients	Age (Mean ± Standard Deviation) or (Median (Range))	Female (N (%))
Aoyama, 2017 [30]	Japan	Case control study	Paclitaxel	Mullerian, endometrial, cervical cancers, and uterine sarcoma	414	Case (HSR group): 44.5 (32–74) Control: 60 (16–83)	-
Ishida, 2020 [34]	Japan	Case control study	Docetaxel	Breast cancer	182	56 (26–78) Case (HSR group): 48 (36–70) Control: 58 (26–78)	182 (100)
Piovano, 2012 [37]	Italy	Case control study	Paclitaxel	Ovarian, endometrial, cervical cancer	157; 101 (Platinum salts), 96 (Paclitaxel)	Case (HSR group): 52.5 ± 11 Control: 59 ± 12.3	-
Rizzo, 2010 [38]	Italy	Case control study	Paclitaxel Docetaxel	Breast cancer	95	57 ± 10	94 (98.9)
Sendo, 2005 [39]	Japan	Case control study	Paclitaxel	Ovarian cancer	105	Case (HSR group): 52.1 ± 11.2 Control: 57.1 ± 11.3	105 (100)
Tangsaghasaks-ri, 2018 [40]	Thailand	Case control study	Paclitaxel	Breast, lung, cervical, ovarian, corpus, endometrial, peritoneal cancers	1132	Case (HSR group): 53.77 ± 10.76 Control: 56.06 ± 11.49	961 (84.9)
Thangwonglers, 2023 [41]	Thailand	Case control study	Paclitaxel	Ovarian, uterine, endometrial, vulvar cancer	416	56.37 ± 10.68	416 (100)
Boso, 2014 [31]	Spain	Retrospective analysis	Paclitaxel Docetaxel	Breast cancer	113; 70 (Docetaxel), 43 (Paclitaxel)	Docetaxel group: 50.5 (48.0–53.0) Paclitaxel group: 57.9 (53.5–62.2)	Docetaxel group: 70 (100) Paclitaxel group: 43 (100)
Haine, 2022 [24]	The Netherlands	Retrospective cohort study	Paclitaxel	Breast, ovarian, lung, endometrial, and miscellaneous cancers	108	60 (52–69)	93 (86.1)
Kwon, 2002 [18]	Canada	Retrospective cohort study	Paclitaxel	Ovarian and primary peritoneal cancer	223	-	-
Lansinger, 2021 [36]	USA (Hispanic 11.88%, Non-Hispanic 85.92%, Unknown 2.2%)	Retrospective cohort study	Paclitaxel Docetaxel	Breast, GI, GU, gynecological, head and neck, lung, thymus, sarcoma and skin cancers	3181	-	2205 (69.32)
Parinyanitikul, 2018 [42]	Thailand	Retrospective cohort study	Paclitaxel	Breast cancer	81	51.0 ± 11 (range 27–74)	81 (100)
Strobbe, 2023 [27]	France	Retrospective analysis	Paclitaxel	Gynecological, breast, head and neck, lung, upper GI, GU cancers, and soft tissue sarcoma,	831	Women: 59.9 ± 13.5 Men: 60.1 ± 14.5	733 (88.2)
Tsang, 2023 [28]	Canada	Retrospective cohort study	Paclitaxel	Breast, ovarian, endometrial, cervical, lung cancers, and others	366	Case (HSR group): 62 (31–84) Control: 63 (25–91)	314 (85.8)
Zwimpfer, 2023 [21]	Switzerland	Retrospective cohort study	Paclitaxel	Gynecological cancers	241; 219 (Platinum salts), 153 (Taxane) *	63 (30.5–85.5) Case (HSR group): 57.1 (40.2–71) Control: 64.9 (30.5–85.5)	-
Gonzalez-Diaz, 2023 [33]	Mexico	Observational descriptive and prospective study	Paclitaxel	Breast, lung, gynecological, GI, and miscellaneous cancers	76	53 ± 12.84	61 (80.3)
Jabir, 2018 [25]	Malaysia (Malay 40%, Chinese 52% and Indian 8%)	Prospective study	Docetaxel	Breast cancer	110	53.66 ± 9.6 (range 31–71)	-
Joly, 2011 [35]	Australia, Belgium, France, Germany, Italy, New Zealand, Sweden, the UK	Post-hoc analysis of data from phase III clinical trial	Paclitaxel	Ovarian cancer	502	61	502 (100)

N: number, HSR: hypersensitivity reaction, USA: United States of America, GI: gastrointestinal, GU: genitourinary, UK, United Kingdom. * 131 patients received concurrent administration of platinum and taxane.

**Table 2:** Summary of taxane hypersensitivity outcomes.

Ref	Types/ Symptoms of HSRs	Chemotherapy Regimens	Evaluated Risk Factors: OR (95% CI) or *p*-Value
[30]	Hypotension, dyspnea, hypoxia, anaphylaxis, chest pain, facial flushing, and generalized urticaria	Weekly/3-weekly PTX + carboplatin, PTX + cisplatin, Weekly PTX alone	Age ≥ 50: 6.31 (2.65–15.7) History of allergy: 3.79 (1.27–10.8) Premedication protocol: 14.1 (1.61–325) Paclitaxel original (vs. generic): 2.56 (0.50–23.3)
[31]	Grade ≥ 2 infusion related reactions graded by NCI CTCAE version 4.0	PTX + monoclonal antibody, PTX alone, PTX + doxorubicin, PTX + doxorubicin + monoclonal antibody, DTX + doxorubicin + cyclophosphamide, DTX + cyclophosphamide, DTX + monoclonal antibody, DTX alone	Genetic polymorphism CYP3A4*1B: *p* = 0.01 CYP3A5*1: *p* = 0.036
[33]	Cutaneous (rash, flushing and pruritus), neuromuscular (low back pain, headache and vision problems), respiratory symptoms (dyspnea, chest tightness and oxygen desaturation) and gastrointestinal symptoms (nausea and abdominal pain), cardiovascular symptoms (hypertension and presyncope), oropharyngeal symptoms (drowning) and general symptoms (chills) The reaction according to Brown’s classification,	PTX alone	Personal history of atomy (PHA) and family history of atopy (FHA): 8 (1.417–43.770) Only PHA: 5 (1.066–24.193) Only FHA: 4.6 (1.016–21.334) Sensitization to environmental allergens: 1 (0.545–1.577) Sensitization to food allergens: 1.513 (0.316–7.255) Sensitization to both allergens: 1.333 (0.139–12.758)
[24]	Hyper-or hypotension, angioedema, flushing, generalized pruritus, hypertension, dyspnea, or chest pain	-	Ranitidine premedication: 1.15 (0.41–3.18)
[34]	Anaphylaxis	DTX alone, DTX + trastuzumab, DTX + cyclophosphamide, DTX + pertuzumab + trastuzumab, DTX + doxorubicin + cyclophosphamide	Age (<48 vs. ≥48) (in dose/administration times analysis): 3.47 (0.94–13.40) Age (<48 vs. ≥48) (in dose/diluent volumes analysis): 3.53 (0.93–13.33) First cycle (in dose/administration times analysis): 2.62 (0.70–9.76) First cycle (in dose/diluent volumes analysis): 2.66 (0.71–9.94) Dose/administration times (>1.15 mg/m^2^/min): 11.60 (2.37–56.79) WBC (<4290 counts/mL) (in dose/administration times analysis): 3.82 (1.06–13.82) WBC (<4290 counts/mL) (in dose/diluent volumes analysis): 3.76 (1.04–13.62) Alb (>4.3 g/dL) (in dose/administration times analysis): 2.87 (0.77–10.74) Alb (>4.3 g/dL) (in dose/diluent volumes analysis): 2.71 (0.75–10.52) Dose/diluent volumes (>0.275 mg/m^2^/mL): 11.88 (2.43–58.16)
[25]	Rash	DTX alone	*ABCB1* 3435C>T Heterozygous: *p* = 0.009
[35]	Allergic reactions graded by NCI CTCAE version 3.0	PTX + carboplatin	Regimen (Carboplatin + Pegylated liposomal doxorubicin vs. Carboplatin + Paclitaxel): 0.252 (0.128–0.495)
[18]	Flushing, chest pain, respiratory distress, hypertension, back pain, pruritis, tachycardia, visual disturbance, hypotension, headache, rash, cardiopulmonary arrest, tonic-clonic seizure, bradycardia, emesis, paresthesia severe hypersensitivity reaction was defined as a combined NCIC-CTG toxicity score_6 or a single life-threatening event with an NCIC-CTG toxicity score of 4	-	Routes of prophylactic corticosteroid (IV vs. PO): 0.44 (0.18–1.07)
[36]	Infusion related reactions graded by NCI CTCAE version 5	-	Total dexamethasone dose *: >20 (mean = 30.8): 0.97 (0.59–1.60), >10–20 (mean = 19.1): 1.06 (0.88–1.28), 0–10 (mean = 9.3): 0.93 (0.69–1.25) Taxane *: Docetaxel: 0.82 (0.64–1.06), Paclitaxel: 1.16 (0.96–1.41) Taxane dose *: High: 1.03 (0.90–1.17), low: 0.96 (0.79–1.17) Route of corticosteroids *: on the day of chemotherapy IV only: 1.12 (0.91–1.38), PO only: 0.79 (0.58–1.06), before chemotherapy: 0.97 (0.60–1.58) Clinical cancer group *: breast: 0.87 (0.68–1.10), GI: 0.77 (0.46–1.29), GU: 1.27 (0.70–2.31), Gynecological: 1.34 (1.01–1.79), head and neck: 0.82 (0.49–1.36), lung: 0.90 (0.57–1.42), sarcoma: 1.31 (0.62–2.79), unknown cancers: 1.72 (0.59–5.023), and others: 1.36 (0.41–4.58) Female sex *: 1.26 (1.09–1.46) Race *: Asian: 1.09 (0.87–1.36), Black: 0.45 (0.17–1.19), White: 1.05 (0.93–1.19), Others: 0.96 (0.73–1.27), Unknown: 0.39 (0.10–1.57)
[42]	Hypersensitivity reactions including flushing and chest discomfort graded by NCI CTCAE version 3	PTX alone ± trastuzumab	Age: *p* = 0.699 Body weight: *p* = 0.606 Obesity (BMI > 30): 0.41 (0.04–3.81), *p* = 0.655 Postmenopausal state: 0.87 (0.14–5.50), *p* = 0.629 Comorbidity: *p* = 0.482 Adjuvant chemotherapy regimen (Paclitaxel alone vs. Paclitaxel + trastuzumab): *p* = 0.568
[37]	Hypersensitivity reaction	PTX + carboplatin, PTX alone, Carboplatin alone, PTX + ifosfamide + platins, and others	Postmenopausal status at the time of chemotherapy: 0.05 (0.01–0.63) Prior history of systemic HSR: 3.42 (0.94–12.45) BMI > 25: 0.68 (0.21–2.21) Age: 0.98 (0.93–1.04) Oral premedication: 1.56 (0.47–5.16)
[38]	Acute dyspnea, flushing of the face, chest constraint, hypotension and rash	-	Age (*p* = 0.1141), Metastasis (*p* = 0.5422), Histotype: ductal, lobular, mixed, apocrine, medullary, micropapillary, metaplastic, poorly differentiated adenocarcinoma (*p* = 0.8410), Genetic polymorphism: *ABCB1* 1236 C>T (*p* = 0.3977), *ABCB1* 2677 G>T/A (*p* = 0.1704), *CYP2C8* 416 G>A (*p* = 0.8204), *CYP2C8* 792 C>G (*p* = 0.1122), *CYP2C8* 1196 A>G (*p* = 0.6858), and *CYP1B1* 4326 C>G (*p* = 0.0015)
[39]	Facial flushing, dyspnea, chest discomfort, hypotension, diaphoresis, hypertension, chest pain, cough, vomiting	PTX + carboplatin	BMI > 25: 8.47 (1.48–48.57) History of mild HSR during the first course: 29.29 (3.99–214.94) Respiratory dysfunction: 10.92 (1.58–75.24) Postmenopausal status at the time of chemotherapy: 5.78 (1.21–27.65)
[27]	Skin rash, dyspnea, back pain, discomfort, hot flush, angioedema, low blood pressure, tachycardia, nausea/vomiting, high blood pressure, chest tightness, epigastralgia, abdominal pain, pelvic pain, headaches, throat irritation	Weekly/3, 4-weekly PTX alone, PTX + carboplatin, PTX + monoclonal antibody, PTX + immune checkpoint inhibitor ± chemotherapy, PTX + carboplatin + monoclonal antibody, PTX + cisplatin or oxaliplatin ± chemotherapy	Types of premedication: famotidine vs. no H2RA: 0.95 (0.18–4.91) Types of premedication: ranitidine vs. no H2RA: 1.21 (0.35–4.21) Types of premedication: H2RA vs. No H2RA: 1.12 (0.36–3.49) Types of premedication: famotidine vs. ranitidine: 0.79 (0.1–4.3) No corticosteroid premedication: 0.08 (0.008–0.78) First three injections: 10.1 (3.23–31.45) Injection cycles: first cycle (vs. ≥4 cycles): 5.14 (1.14–23.2) second cycle (vs. ≥4 cycles): 16.7 (4.4–62.8) third cycle (vs. ≥4 cycles): 10.6 (2.6–42.7)
[40]	Flushing, dyspnea, pruritis, hypertension, tachycardia, bronchospasm, hypotension, anaphylaxis, nausea vomiting, abdominal pain determined in accordance with NCI CTCAE v4.03.	-	Age: *p* = 0.177 BMI: *p* = 0.937 Serum creatinine: *p* = 0.630 Paclitaxel dosage: *p* = 0.981 Female sex: 1.12 (0.36–3.49) Marital status: *p* = 0.269 Allergy history: 1.66 (0.79–3.51) Career (Agriculture vs. Non-agriculture): 2.25 (1.20–5.43) Underlying disease (Yes vs. No): *p* = 0.137
[41]	Flushing, pruritis, maculopapular rash, angioedema, nausea, vomiting, discomfort, pain (chest, abdominal, back, headache), hypotension, hypertension, tachycardia, anaphylaxis graded by NCI CTCAE version 4.03 or 5.0	PTX + carboplatin, PTX + cisplatin, PTX alone, PTX + ifosfamide, PTX + carboplatin + bevacizumab	Age (<54.5 years): 2.35 (1.405–3.932) History of allergy: 0.953 (0.511–1.779) Ovarian cancer: 0.902 (0.545–1.493) BMI > 25: 0.841 (0.498–1.422) Cancer stage ≥ 2: 1.846 (1.001–3.402) Asthma: 0.507 (0.063–4.063) WBC < 7.735: 2.22 (1.311–3.765) Absolute eosinophil count ≥ 0.615: 1.008 (0.213–4.765) Postmenopausal status at the time of chemotherapy: 0.481 (0.273–0.847)
[28]	Infusion related reactions graded by NCI CTCAE	-	No H2RA premedication: 1.26 (0.69–2.28)
[21]	Skin, respiratory, GI, cardiovascular, and other symptoms	Taxane + platins	Age (continuous variable): *p* = 0.030 Gynecologic cancer: *p* = 0.056 Ethnicity: Caucasian, Hispanic, and Asian (*p* = 0.472) Family history of gynecologic cancer: *p* = 0.782 Concurrent platinum chemotherapy: *p* = 0.118 Lines of chemotherapy: *p* = 0.552 Cycles of chemotherapy: *p* = 0.164 Cumulative dose of platinum: *p* = 0.548

Alb: albumin, BMI: body mass index, CI: confidence interval, DTX: docetaxel, GI: gastrointestinal, GU: genitourinary, H2RA: histamine 2 receptor antagonist, HSR: hypersensitivity reaction, IV: intravenous, NCI CTCAE: National Cancer Institute Common Terminology Criteria for Adverse Events (CTCAE), NCIC-CTG: National Cancer Institute of Canada-Clinical Trials Group, OR: odds ratio, PO: per oral, PTX: paclitaxel, Ref: reference, WBC: white blood cell. * hazard ratio (95% CI).

## Data Availability

The data generated during and/or analyzed during the current study are available from the corresponding author on reasonable request. Requests to access the dataset should be directed to J.M.O., jmoh@snu.ac.kr.

## Supplementary Materials

The following supporting information can be downloaded at: https://www.mdpi.com/article/10.3390/jpm15010002/s1, Supplementary S1: Patient, Intervention, Comparator, Outcome (PICO) Framework and search strategy; Supplementary S2: Definitions of risk factors in studies included in the meta-analysis; Supplementary S3: Risk of bias assessment (Newcastle-Ottawa Scale (NOS)); Supplementary S4: Publication bias assessment—funnel plot (A), (B); Supplementary S5: Sensitivity analysis: results of leave-one-out analysis. (A) History of allergy; (B) Obesity; (C) Postmenopausal state.
