# Supplementary material for: Personalized Risk Assessment for Taxane-Induced Hypersensitivity Reactions: A Systematic Review and Meta-Analysis
Source: J Pers Med. 2024 Dec 24;15(1):2. doi: 10.3390/jpm15010002 (PMC11767215; doi:10.3390/jpm15010002)
Supplement: Supplementary file 1 [file jpm-15-00002-s001.zip › jpm-3302939-supplementary.pdf]

Supplementary

- **Supplementary S1: Patient, Intervention, Comparator, Outcome (PICO) Framework and search strategy**
- **Supplementary S2: Definitions of risk factors in studies included in the meta-analysis**
- **Supplementary S3: Risk of bias assessment (Newcastle-Ottawa Scale (NOS))**
- **Supplementary S4: Publication bias assessment – funnel plot (A), (B)**
- **Supplementary S5: Sensitivity analysis: results of leave-one-out analysis (A) History of allergy (B) Obesity (C) Postmenopausal state**

Supplementary S1: Patient, Intervention, Comparator, Outcome (PICO) Framework and search strategy

Patient, Intervention, Comparator, Outcome (PICO) Framework

**Framework (PICO)**	**Description**	**Concept**
POPULATION	Adult patients (age>18) administered taxane-containing chemotherapy regimens	Docetaxel<br>Paclitaxel<br>Cabazitaxel<br>Taxanes<br>Adult<br>Cancer
INTERVENTION	Genetic polymorphisms, variants, or risk factors	Risk factor<br>Genotype<br>Allele<br>Polymorphism<br>Pharmacogenetic factor
COMPARATOR	-	-
OUTCOME	Taxane-induced hypersensitivity reactions, including infusion related reactions, allergic reactions, anaphylaxis, and severe cutaneous adverse reactions (SCARs)	Hypersensitivity reaction<br>Infusion-related reaction<br>Allergy<br>Anaphylaxis reaction<br>Severe cutaneous adverse reaction (SCAR)<br>Stevens-Johnson syndrome (SJS)<br>Drug reaction with eosinophilia systemic symptoms (DRESS)<br>Toxic epidermal necrolysis (TEN)

Search strategy for each literature database

(Search query for MEDLINE)

1. Species: Human
2. Article language: English
3. (docetaxel* or paclitaxel* or cabazitaxel* or taxan*).ti.
4. (risk* or factor* or gene* or genotyp* or allele* or polymorphism* or pharmacogene* or variant* or haplotype* or genome* or SNP or HLA).ab,ti.
5. (hypersensitivit* or HSR or infusion related* or infusion reaction* or allerg* or anaphyla* or SCAR* or toxic epidermal necrolysis* or SJS or stevens johnson syndrome* or DRESS* or drug reaction with eosinophilia systemic symptoms*).ab,ti.
6. 3 AND 4 AND 5

(Search query for PubMed)

1. Species: Human
2. Article language: English
3. ("docetaxel"[Title] OR "paclitaxel"[Title] OR "cabazitaxel"[Title] OR "taxan*"[Title])
4. ("risk*"[Title/Abstract] OR "factor*"[Title/Abstract] OR ("gene*"[Title/Abstract] OR "genotyp*"[Title/Abstract] OR "allele*"[Title/Abstract] OR "polymorphism*"[Title/Abstract] OR "pharmacogene*"[Title/Abstract] OR "variant*"[Title/Abstract] OR "haplotype*"[Title/Abstract] OR "genome*"[Title/Abstract] OR "SNP"[Title/Abstract] OR "HLA"[Title/Abstract]))
5. ("hypersensitivit*"[Title/Abstract] OR "HSR"[Title/Abstract] OR "infusion related*"[Title/Abstract] OR "infusion reaction*"[Title/Abstract] OR "allerg*"[Title/Abstract] OR "anaphyla*"[Title/Abstract] OR "SCAR*"[Title/Abstract] OR "toxic epidermal necrolysis*"[Title/Abstract] OR "SJS"[Title/Abstract] OR "stevens johnson syndrome*"[Title/Abstract] OR "DRESS*"[Title/Abstract] OR "drug reaction with eosinophilia systemic symptoms*"[Title/Abstract])
6. 3 AND 4 AND 5

(Search query for EMBASE)

1. Species: Human
2. Article language: English
3. docetaxel*:ti OR paclitaxel*:ti OR cabazitaxel*:ti OR taxan*:ti
4. risk*:ab,ti OR factor*:ab,ti OR gene*:ab,ti OR genotyp*:ab,ti OR allele*:ab,ti OR polymorphism*:ab,ti OR pharmacogene*:ab,ti OR variant*:ab,ti OR haplotype*:ab,ti OR genome*:ab,ti OR snp:ab,ti OR hla:ab,ti
5. hypersensitivit*:ab,ti OR hsr:ab,ti OR 'infusion related*':ab,ti OR 'infusion reaction*':ab,ti OR allerg*:ab,ti OR anaphyla*:ab,ti OR scar*:ab,ti OR 'toxic epidermal necrolysis*':ab,ti OR sjs:ab,ti OR 'stevens johnson syndrome*':ab,ti OR dress*:ab,ti OR 'drug reaction with eosinophilia systemic symptoms*':ab,ti
6. 3 AND 4 AND 5

(Search query for Web of Science)

1. Species: Human
2. Article language: English
3. (docetaxel* or paclitaxel* or cabazitaxel* or taxan*) title
4. (risk* or factor* or gene* or genotyp* or allele* or polymorphism* or pharmacogene* or variant* or haplotype* or genome* or SNP or HLA) (title) OR (risk* or factor* or gene* or genotyp* or allele* or polymorphism* or pharmacogene* or variant* or haplotype* or genome* or SNP or HLA) (abstract)
5. (hypersensitivit* or HSR or infusion related* or infusion reaction* or allerg* or anaphyla* or SCAR* or toxic epidermal necrolysis* or SJS or stevens johnson syndrome* or DRESS* or drug reaction with eosinophilia systemic symptoms*) (title) OR (hypersensitivit* or HSR or infusion related* or infusion reaction* or allerg* or anaphyla* or SCAR* or toxic epidermal necrolysis* or SJS or stevens johnson syndrome* or DRESS* or drug reaction with eosinophilia systemic symptoms*) (abstract)
6. 3 AND 4 AND 5

Supplementary S2: Definitions of risk factors in studies included in the meta-analysis

History of allergy

Study name	Definition of risk factor
Aoyama, 2017 [1]	Previous allergic reaction caused by medical drugs and/or contrast media
Thangwonglers, 2023 [2]	Previous allergic reaction caused by medication, environmental factors, foods, contrast media
Tangsaghasaksri, 2018 [3]	NS
Piovano, 2012 [4]	History of systemic hypersensitivity to drugs, environmental or animal exposure

NS: Not stated

Obesity

Study name	Definition of risk factor
Piovano, 2012 [4]	BMI > 25 kg/m^2
Sendo, 2005 [5]	BMI > 25 kg/m^2
Thangwonglers, 2023 [2]	BMI > 25 kg/m^2
Parinyanitikul, 2018 [6]	BMI > 25 kg/m^2

Postmenopausal state

Study name	Definition of risk factor
Piovano, 2012 [4]	Menopausal status at treatment start time
Sendo, 2005 [5]	Postmenopausal at the time of ovariectomy
Thangwonglers, 2023 [2]	Menopausal status recorded before receiving chemotherapy
Parinyanitikul, 2018 [6]	NS

NS: Not stated

Ovarian cancer

Study name	Definition of risk factor
Thangwonglers, 2023 [2]	Cancer indicated for paclitaxel-based chemotherapy
Tsang, 2023 [7]	Primary tumor site indicated for paclitaxel-based chemotherapy

H2 receptor antagonist premedication

Study name	Definition of risk factor
Strobbe, 2023 [8]	Ranitidine and famotidine administration before paclitaxel infusion
Haine, 2022 [9]	Ranitidine administration before paclitaxel infusion
Tsang, 2023 [7]	Ranitidine and famotidine administration before paclitaxel infusion

Supplementary S3: Risk of bias assessment (Newcastle-Ottawa Scale (NOS)) [1–18]

Criteria	Selection (Total 4 points)				Comparability (Total 2 points)	Outcome (Total 3 points)			Score	Quality
Study	Representativeness of the Exposed Cohort	Selection of the Non-Exposed Cohort	Ascertainment of Exposure	Demonstration that Outcome of Interest Was Not Present at Start of Study	Comparability of Cohorts on the Basis of the Design or Analysis	Assessment of Outcome	Was Follow-up Long Enough for Outcomes to Occur?	Adequacy of Follow-up of Cohorts		
Aoyama, 2017	a*	a*	a*	b	b*	b*	a*	a*	7	Moderate
Boso, 2014	a*	b	a*	a*	a	b*	b	d	6	Moderate
Gonzalez-Diaz, 2023	a*	a*	b*	a*	b*	b*	a*	d	7	Moderate
Haine, 2022	b*	a*	a*	a*	a	b*	a*	d	8	Good
Ishida, 2020	a*	a*	b	a*	a*	a*	a*	a*	8	Good
Jabir, 2018	b*	a*	a*	a*	b*	a*	b	d	6	Moderate
Joly, 2011	a*	a*	a*	a*	a*	a*	a*	d	8	Good
Kwon, 2002	a*	a*	a*	a*	a*	b*	a*	a*	9	Good
Lansinger, 2021	a*	a*	a*	a*	b*	b*	a*	a*	8	Good
Parinyanitikul, 2018	a*	a*	a*	a*	b*	a*	a*	a*	8	Good
Piovano, 2012	a*	a*	a*	a*	a*	b*	a*	a*	9	Good
Rizzo, 2010	a*	a*	a*	a*	a*	b*	a*	a*	9	Good
Sendo, 2005	a*	a*	a*	a*	b*	b*	a*	a*	8	Good
Strobbe, 2023	a*	a*	a*	a*	b*	b*	a*	a*	8	Good
Tangsaghasaksri, 2018	a*	a*	a*	a*	b*	b*	a*	a*	8	Good
Thangwonglers, 2023	a*	a*	a*	a*	b*	b*	a*	a*	8	Good
Tsang, 2023	b*	a*	a*	a*	b*	b*	a*	a*	8	Good
Zwimpfer, 2023	a*	a*	a*	b	b*	b*	a*	a*	7	Good

Supplementary S4: Publication bias assessment – funnel plot (A), (B)

(A) History of allergy

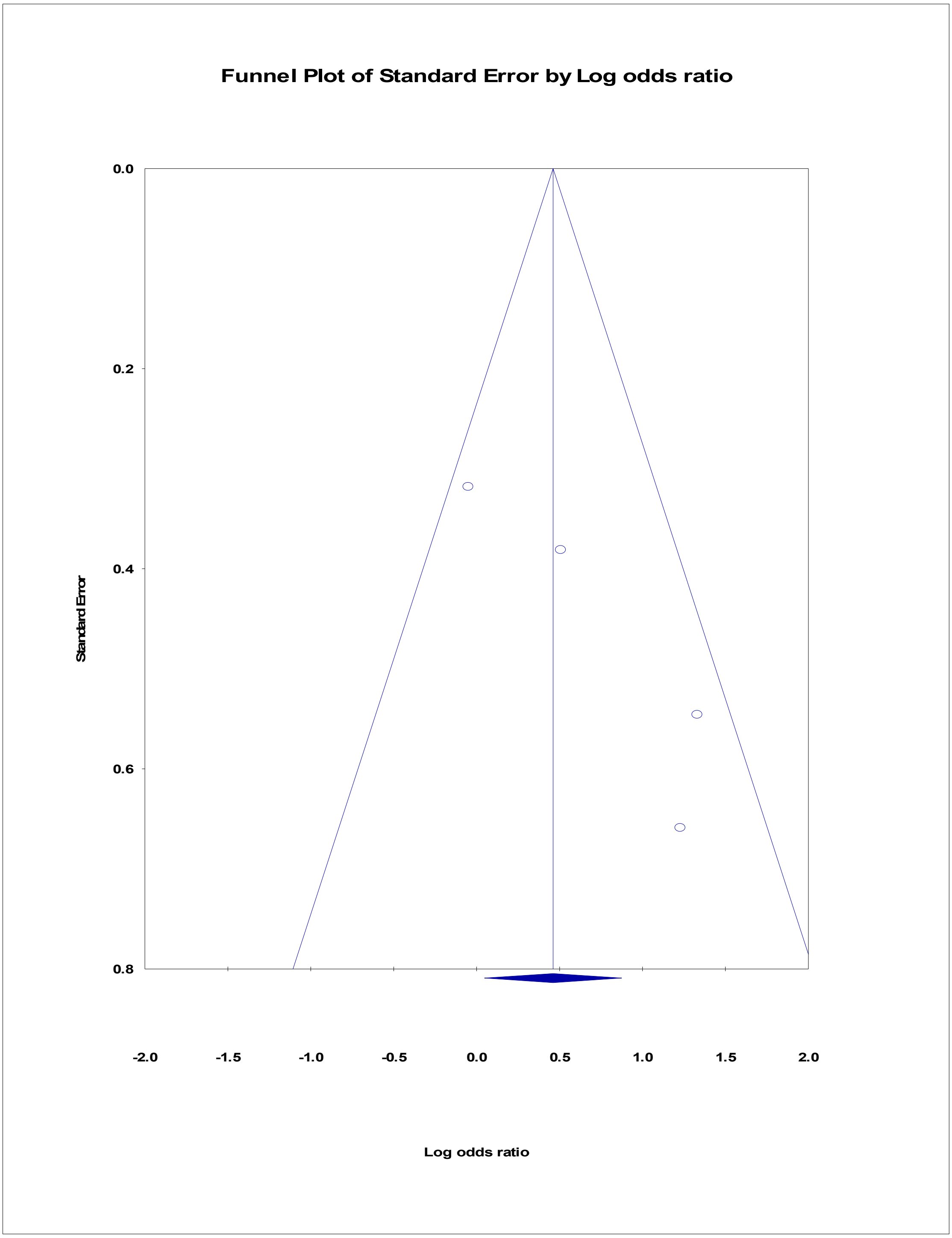

(B) Obesity

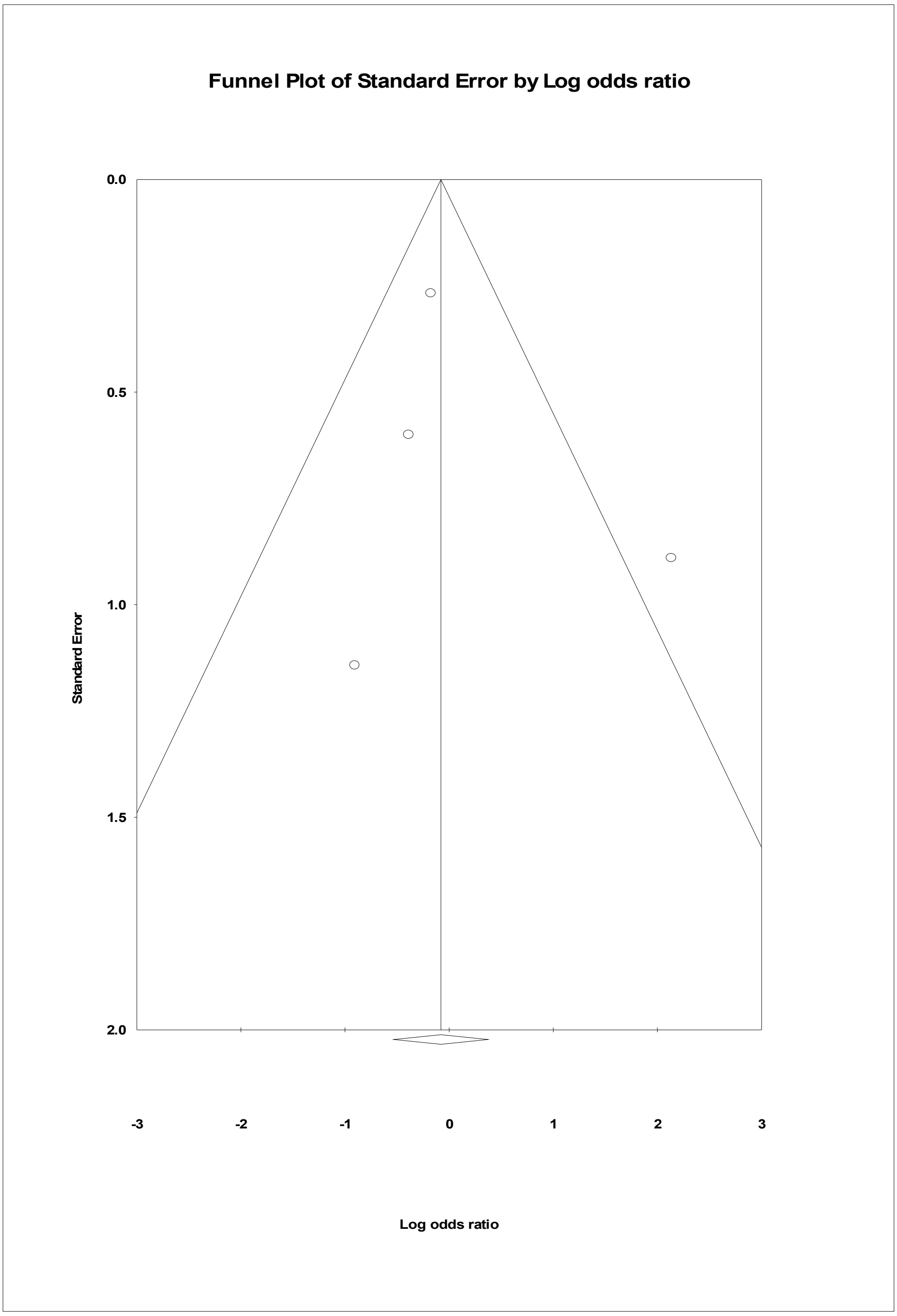

Funnel Plot of Standard Error by Log odds ratio
0.0
0.5
1.0
1.5
2.0
Standard Error
-3
-2
-1
0
1
2
3
Log odds ratio

Supplementary S5: Sensitivity analysis: results of leave-one-out analysis (A) History of allergy (B) Obesity (C) Postmenopausal state [1,2,4,6]

A — **History of allergy**

Study name	Odds ratio	Lower limit	Upper limit	Z-Value	P-Value
Aoyama, 2017	3.79	1.30	11.05	2.44	0.01
Thangwonglers, 2023	0.95	0.51	1.78	-0.15	0.88
Piovano, 2012	3.42	0.94	12.45	1.87	0.06
Pooled	2.09	0.76	5.74	1.43	0.15

(Statistics for each study)

Heterogeneity: $\tau^2 = 0.13$; Q-value = 3.49; P = 0.18; $I^2 = 42.6\%$

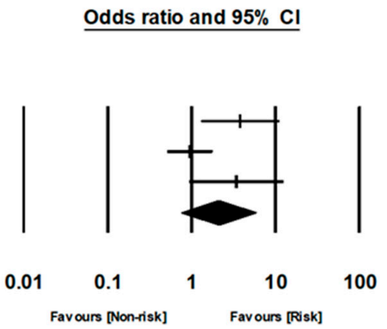

B — **Obesity**

Study name	Odds ratio	Lower limit	Upper limit	Z-Value	P-Value
Piovano, 2012	0.68	0.21	2.21	-0.64	0.52
Thangwonglers, 2023	0.84	0.50	1.42	-0.65	0.52
Parinyanitikul, 2018	0.41	0.04	3.81	-0.79	0.43
Pooled	0.79	0.49	1.26	-1.00	0.32

Heterogeneity: $\tau^2 < 0.01$; Q-value = 0.46; P = 0.80; $I^2 < 0.1\%$

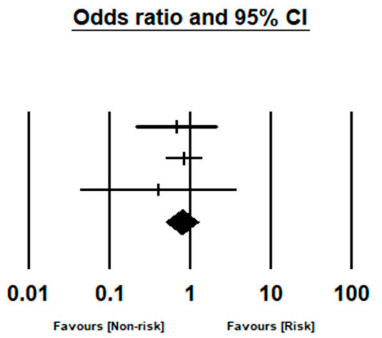

C — **Postmenopausal state**

Study name	Odds ratio	Lower limit	Upper limit	Z-Value	p-Value
Piovano, 2012	0.05	0.01	0.40	-2.83	0.00
Thangwonglers, 2023	0.48	0.27	0.85	-2.53	0.01
Parinyanitikul, 2018	0.87	0.14	5.50	-0.15	0.88
Pooled	0.33	0.09	1.22	-1.66	0.10

(Statistics for each study)

Heterogeneity: $\tau^2 = 0.79$; Q-value = 4.85; P = 0.09; $I^2 = 58.7\%$

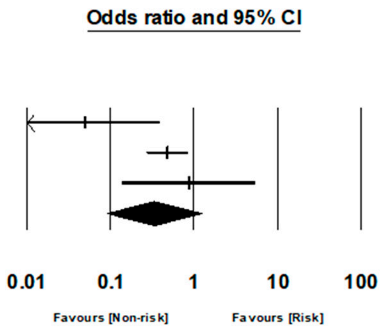

CI: confidence interval

References

1. Aoyama, T.; Takano, M.; Miyamoto, M.; Yoshikawa, T.; Soyama, H.; Kato, K.; Ishibashi, H.; Iwahashi, H.; Nakatsuka, M.; Yajima, I.; et al. Is there any predictor for hypersensitivity reactions in gynecologic cancer patients treated with paclitaxel-based therapy? *Cancer Chemother. Pharmacol.* **2017**, *80*, 65–69. https://doi.org/10.1007/s00280-017-3332-7.
2. Thangwonglers, T.; Santimaleeworagun, W.; Therasakvichya, S.; Saengsukkasemsak, N.; Pimsi, P. Characteristics of immediate hypersensitivity reaction to paclitaxel-based chemotherapy in gynecologic cancer patients. *Asian Pac. J. Allergy Immunol.* **2023**, *41*, 340–346. https://doi.org/10.12932/AP-050520-0831.
3. Tangsaghasaksri, R.; Jainan, P. Incidence of Hypersensitivity Reactions from Paclitaxel. *J. Med. Assoc. Thai.* **2018**, *101*, S150–S154.
4. Piovano, E.; Pivetta, E.; Modaffari, P.; Martra, F.; Baima Poma, C.; Perotto, S.; Tripodi, E.; Zanfagnin, V.; Zola, P.; Ferrero, A. A search for predictive factors for hypersensitivity reactions to paclitaxel and platinum salts in chemotherapy for gynecologic pelvic neoplasms. *Gynecol. Obstet. Invest.* **2012**, *74*, 21–27. https://doi.org/10.1159/000336772.
5. Sendo, T.; Sakai, N.; Itoh, Y.; Ikesue, H.; Kobayashi, H.; Hirakawa, T.; Nakano, H.; Oishi, R. Incidence and risk factors for paclitaxel hypersensitivity during ovarian cancer chemotherapy. *Cancer Chemother. Pharmacol.* **2005**, *56*, 91–96. https://doi.org/10.1007/s00280-004-0924-9.
6. Parinyanitikul, N.; Tanpipattanakul, W.; Poovorawan, N.; Rattananupong, T.; Laoitthi, P.; Sithidetphaiboon, P.; Thanasanvimon, S.; Sriuranpong, V. Incidence of infusion hypersensitivity reaction after withholding dexamethasone premedication in early breast cancer patients not experiencing two previous cycles of infusion hypersensitivity reaction for weekly paclitaxel chemotherapy. *Support. Care Cancer* **2018**, *26*, 2471–2477. https://doi.org/10.1007/s00520-018-4087-3.
7. Tsang, C.; Robinson, J.; Wheatley-Price, P.F.; Brule, S.Y.; Moore, S.M. The utility of H2 receptor antagonists in preventing infusion-related reactions to paclitaxel chemotherapy. *Cancer* **2023**, *129*, 3815–3819. https://doi.org/10.1002/cncr.35006.
8. Strobbe, G.; Gaboriau, L.; Abele, M.; Villain, A.; Aelbrecht-Meurisse, C.; Carnot, A.; Le Deley, M.C.; Leguillette, C.; Feutry, F.; Sakji, I.; et al. Impact of histamine-2 antagonist shortage on the incidence of hypersensitivity reactions to paclitaxel: A reconsideration of premedication protocols in France (PACLIREACT Study). *Eur. J. Clin. Pharmacol.* **2023**, *79*, 1229–1238. https://doi.org/10.1007/s00228-023-03536-x.
9. Haine, A.I.; Notenboom, C.; Tan, L.V.P.; Ruiter, R.; van der Deure, W.M. Ranitidine and the incidence of hypersensitivity reactions to paclitaxel: A retrospective cohort study. *Pharmacol. Res. Perspect.* **2022**, *10*, e00985. https://doi.org/10.1002/prp2.985.
10. Boso, V.; Herrero, M.J.; Santaballa, A.; Palomar, L.; Megias, J.E.; de la Cueva, H.; Rojas, L.; Marques, M.R.; Poveda, J.L.; Montalar, J.; et al. SNPs and taxane toxicity in breast cancer patients. *Pharmacogenomics* **2014**, *15*, 1845–1858. https://doi.org/10.2217/pgs.14.127.
11. Gonzalez-Diaz, S.N.; Canel-Paredes, A.; Macias-Weinmann, A.; Vidal-Gutierrez, O.; Villarreal-Gonzalez, R.V. Atopy, allergen sensitization and development of hypersensitivity reactions to paclitaxel. *J. Oncol. Pharm. Pract.* **2023**, *29*, 810–817. https://doi.org/10.1177/10781552221080415.
12. Ishida, S.; Masuguchi, K.; Kawashiri, T.; Tsuji, T.; Watanabe, H.; Akiyoshi, S.; Kubo, M.; Masuda, S.; Egashira, N. Effects of Diluent Volume and Administration Time on the Incidence of Anaphylaxis Following Docetaxel Therapy in Breast Cancer. *Biol. Pharm. Bull.* **2020**, *43*, 663–668. https://doi.org/10.1248/bpb.b19-00876.
13. Jabir, R.S.; Ho, G.F.; Annuar, M.; Stanslas, J. Association of Allelic Interaction of Single Nucleotide Polymorphisms of Influx and Efflux Transporters Genes With Nonhematologic Adverse Events of Docetaxel in Breast Cancer Patients. *Clin. Breast Cancer* **2018**, *18*, e1173–e1179. https://doi.org/10.1016/j.clbc.2018.04.018.
14. Joly, F.; Ray-Coquard, I.; Fabbro, M.; Donoghoe, M.; Boman, K.; Sugimoto, A.; Vaughan, M.; Reinthaller, A.; Vergote, I.; Ferrandina, G.; et al. Decreased hypersensitivity reactions with carboplatin-pegylated liposomal doxorubicin compared to carboplatin-paclitaxel combination: Analysis from the GCIG CALYPSO relapsing ovarian cancer trial. *Gynecol. Oncol.* **2011**, *122*, 226–232. https://doi.org/10.1016/j.ygyno.2011.04.019.

15. Kwon, J.S.; Elit, L.; Finn, M.; Hirte, H.; Mazurka, J.; Moens, F.; Trim, K. A comparison of two prophylactic regimens for hypersensitivity reactions to paclitaxel. *Gynecol. Oncol.* **2002**, *84*, 420–425. https://doi.org/10.1006/gyno.2001.6546.
16. Lansinger, O.M.; Biedermann, S.; He, Z.; Colevas, A.D. Do Steroids Matter? A Retrospective Review of Premedication for Taxane Chemotherapy and Hypersensitivity Reactions. *J. Clin. Oncol.* **2021**, *39*, 3583–3590. https://doi.org/10.1200/JCO.21.01200.
17. Rizzo, R.; Spaggiari, F.; Indelli, M.; Lelli, G.; Baricordi, O.R.; Rimessi, P.; Ferlini, A. Association of CYP1B1 with hypersensitivity induced by taxane therapy in breast cancer patients. *Breast Cancer Res. Treat.* **2010**, *124*, 593–598. https://doi.org/10.1007/s10549-010-1034-5.
18. Zwimpfer, T.A.; Scherer, K.; Schotzau, A.; Heinzelmann-Schwarz, V.; Hartmann, K.; Vetter, M.; Montavon, C. Desensitization in patients with hypersensitivity to platinum and taxane in gynecological cancers. *Cancer. Med.* **2024**, *13*, e6840. https://doi.org/10.1002/cam4.6840.
